# Evaluation of Non-Local Means Based Denoising Filters for Diffusion Kurtosis Imaging Using a New Phantom

**DOI:** 10.1371/journal.pone.0116986

**Published:** 2015-02-02

**Authors:** Min-Xiong Zhou, Xu Yan, Hai-Bin Xie, Hui Zheng, Dongrong Xu, Guang Yang

**Affiliations:** 1 Shanghai Key Laboratory of Magnetic Resonance, Physics Department, East China Normal University, Shanghai, China; 2 Shanghai Medical Instrumentation College, University of Shanghai for Science and Technology, Shanghai, China; 3 MR Collaboration NE Asia, Siemens Healthcare, Shanghai, China; 4 Key Laboratory of Brain Functional Genomics, Institute of Cognitive Neuroscience, East China Normal University, Shanghai, China; 5 MRI Unit, and Epidemiology Division, Columbia University Department of Psychiatry, and New York State Psychiatric Institute, NYSPI Unit 24, 1051 Riverside Drive, New York, New York, 10032, United States of America; Institution of Automation, CAS, CHINA

## Abstract

Image denoising has a profound impact on the precision of estimated parameters in diffusion kurtosis imaging (DKI). This work first proposes an approach to constructing a DKI phantom that can be used to evaluate the performance of denoising algorithms in regard to their abilities of improving the reliability of DKI parameter estimation. The phantom was constructed from a real DKI dataset of a human brain, and the pipeline used to construct the phantom consists of diffusion-weighted (DW) image filtering, diffusion and kurtosis tensor regularization, and DW image reconstruction. The phantom preserves the image structure while minimizing image noise, and thus can be used as ground truth in the evaluation. Second, we used the phantom to evaluate three representative algorithms of non-local means (NLM). Results showed that one scheme of vector-based NLM, which uses DWI data with redundant information acquired at different b-values, produced the most reliable estimation of DKI parameters in terms of Mean Square Error (MSE), Bias and standard deviation (Std). The result of the comparison based on the phantom was consistent with those based on real datasets.

## Introduction

Diffusion kurtosis imaging (DKI) [1] is a new in vivo method for diffusion imaging that originated from diffusion tensor imaging (DTI) [2]. DTI provides a way for probing the microstructure of biological tissues by measuring the diffusion coefficient of water molecules using Gaussian models. Gaussian models can only be used to depict free diffusion processes [1]. However, water diffusion in biological tissues is normally restricted, which constitutes the basis of DTI tractography, and therefore is not exactly Gaussian. To address this self-contradiction, DKI is proposed to model the Gaussian coefficient of diffusion as well as the deviation from the Gaussian model, thereby providing new insights into the microstructures [3], [4], [5], [6], [7]. DKI has already been successfully used in a wide range of clinical studies, including studies in Parkinson’s disease [8], Huntington’s disease [9], epilepsy [10], aging [11], attention deficit hyperactivity disorder [12] and cerebral gliomas [13].

DTI data are composed of baseline images without applying a diffusion gradient and a series of diffusion-weighted (DW) images with diffusion gradients applied along different directions. Typically, DW images are acquired at one b-value, which is an index calculated based on the strength and duration of the diffusion gradient field, and the interval between the two opposite gradients. The diffusion coefficient along a certain direction can be calculated using the following equation:

$${\text{S}}_{\text{n}}=S_{0}\exp(-bD_{n})$$ (1)

where *S*_*0*_ is the baseline signal, *S*_*n*_ is the DW image acquired with diffusion gradient along the n^th^ direction, b is the b-value of the applied diffusion gradient and *D*_*n*_ is the diffusion coefficient along the n^th^ direction. To construct a diffusion tensor, at least seven DW measurements are needed, including a baseline image.

In DTI, only one DW image is needed for each direction. However, DKI requires multiple DW images at different b-values along each direction [1]. DKI uses the following equation to depict the change of the signal intensity with respect to b-values:

$$S_{n}(b)=S_{0}\exp(-bD_{n}+b^{2}D_{n}^{2}K_{n}/6)$$ (2)

where *S*_*0*_, *S*_*n*_, b, and *D*_*n*_ remain the same as in *Eq*. (1), and *K*_*n*_ is the kurtosis coefficient, which depicts the deviation from a Gaussian model. For each direction, both *D*_*n*_ and *K*_*n*_ can be calculated by curve fitting using the baseline image and the corresponding DW images. Then mean diffusion (MD) and mean kurtosis (MK) values can be calculated by averaging the *D*_*n*_ and *K*_*n*_ along each direction [1]. The MD and MK can also be calculated from diffusion and kurtosis tensor respectively, which are reconstructed using *D*_*n*_ and *K*_*n*_ along all directions [5]. The two methods generate similar MD and MK values, while the direct averaging method makes no restriction on the number of diffusion gradient directions and therefore is more efficient in computation [14].

To profile the kurtosis deviation, a typical DKI setting would employ the maximum b-value from 2000 to 2500 s/mm^2^ [1], which is much higher than the 1000 s/mm^2^ that is typically used in DTI. DW images in a DKI dataset therefore often suffer from heavier noise, resulting in low signal-to-noise ratios (SNR) at these higher b-values. The noise, which is Rician [15], [16], [17], in turn, may significantly affect the reliability of parameter estimation. A previous study showed that Rician noise in DW images may lead to significant overestimation of the kurtosis coefficients [15].

Various denoising methods have been developed to improve the quality of DW images, such as the Gaussian filter [11], [18], anisotropic diffusion filter [19], [20], [21], [22], linear minimum mean squared error filter [23], and non-local means (NLM) filter [24]. The NLM filter outperforms most other filters in both denoising and edge preserving, and thus has been used extensively in magnetic resonance (MR) image denoising [25], [26] [27] [28]. While DW images are often denoised on an image-wise basis, correlation between DW images should also be exploited, as it contains spatial cues of the imaging data. Wiest et al. proposed a vector non-local means (VNLM) filter based on the NLM filter for denoising DTI data [29]. It bundles all DW images in a DTI dataset into a vector image and applies NLM to denoise it as one whole entity.

To identify the best denoising algorithm among the existing algorithms or to further tailor them for treating a specific type of image, a method to assess their performance is required. Both visual [23], [24], [29], [30] and quantitative [25], [29], [31], [32] comparisons can be used for this purpose. In a visual comparison, a good denoising algorithm should have (1) minimized image noise; (2) preserved image details; and (3) introduced no artifacts. Quantitative comparisons often use noise-free images as ground truth, to which noise of a known distribution is added for simulating phantom data for testing purposes. In this way, a denoised image can easily be compared quantitatively with the ground truth. Because DTI and DKI contain high dimensional information in each voxel, ground truth based on the maps of diffusion-derived parameters is often favorable for evaluating the performance of denoising algorithms for diffusion imaging data. In previous studies of DTI denoising, DTI phantoms were constructed for denoising DTI data by evaluating the reliability of estimating DTI-derived parameters [33] [34] [35]. However, to the best of our knowledge, no such phantom has been reported for DKI denoising, although various DKI schemes were previously evaluated systematically using a specifically designed simulation dataset of diffusion parameters [36].

To evaluate how denoising algorithms can affect the precision of DKI parameter estimation, we developed a pipeline for constructing DKI phantoms and consequently created a DKI phantom from real brain data. We used it to quantitatively evaluate NLM and two different VNLM schemes. The first VNLM scheme combined DW images at the same b-value as a vector whereas the second combined DW images along the same diffusion gradient direction as a vector. We conducted evaluations using our phantom to check which VNLM performs the best.

## Materials and Methods

### Materials

This study was approved by the Institutional Ethics Committee of East China Normal University. Four local volunteers were recruited to the study and informed written consents were obtained from all these volunteers. A 12-channel head coil was used in data acquisition. DKI data from the volunteers were collected on a 3T Siemens Trio system (maximum gradient strength = 40 mT/m, maximum slew rate = 200 mT/m/ms). A bipolar single-shot EPI sequence [37] was used for DW image acquisition to minimize the eddy current artifacts. Dataset 1, which contained DKI data from one volunteer, was acquired using conventional acquisition parameters for DKI data, with DW images at 6 b-values (0, 500, 1000, 1500, 2000, 2500 s/mm^2^) along 30 diffusion gradient directions, 35 slices, NEX = 2 (averaged in image domain), spatial resolution = 2 × 2 × 3 mm^3^, FOV = 256 × 256 mm^2^, acceleration factor of parallel imaging = 2 (GRAPPA). The other parameters were: TR / TE = 6000 ms / 112 ms, diffusion time Δ = 39.1 ms, and diffusion gradient duration δ = 37.5 ms. The diffusion time Δ here is defined not exactly the same as that in standard Stejskal–Tanner (monopolar) sequence because we used the bipolar single-shot EPI sequence here, while Δ has been calculated according to the conventional expression b = -(γGδ)^2^ (Δ – δ/3) [1]. The acquisition time was 30 min 32 sec. Thirty extra baseline images (therefore a total of 32 baseline images) were collected for generating one baseline image of high SNR. This dataset was later used for DKI phantom construction. Dataset 2 contained DKI data from the other three volunteers that were acquired with slightly different parameters. DW images were collected along 12 / 12 / 20 diffusion gradient directions, TR = 6100 / 12000 / 5300 ms, TE = 114 ms, Δ = 40.1 ms, δ = 38.5 ms, 35 / 40 / 40 slices, and NEX = 1. The other parameters were the same as those used for Dataset 1. In addition, we applied physical constraints to the participants during the data acquisition, and screened the acquired data afterwards to prevent motion-induced artifacts. All the raw data are available at http://pan.baidu.com/s/1ntMD68x#path=%252F.

### DKI Phantom Construction

**Fig 1:**
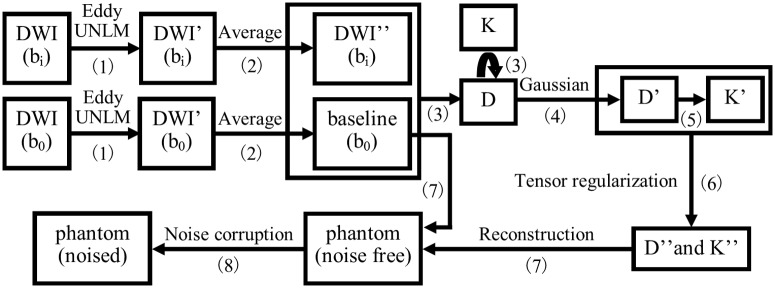
The pipeline for constructing DKI phantoms.

Because no noise-free DKI image is readily available as ground truth, we developed a pipeline to construct a DKI phantom based on a real DKI dataset of the human brain (Fig. 1).

The process consists of 8 steps: **(1)** denoise each DW image (non-brain region was removed through the BET tool of FSL (http://www.fmrib.ox.ac.uk/fsl/)) using a 3D UNLM filter (see next subsection) after eddy current correction and motion correction using ACID Toolbox (http://www.diffusiontools.com); **(2)** average the DW images from repeated scans; **(3)** estimate D and K value maps for each gradient direction using *Eq*. (2); **(4)** apply 3D Gaussian filter to D maps using a Gaussian kernel of 2 mm *full-width-at-half-maximum* (FWHM), and consequently obtain D’; **(5)** recalculate K’ with obtained D’ using *Eq*. (2); note that in step 4) and 5), we do not directly smooth K but recalculate K using a smoothed D as the noise in the D map may significantly influence K estimation, especially for voxels with small D values [14]; **(6)** reconstruct diffusion tensors and kurtosis tensors using the D’ and K’ maps of all gradient directions, and the tensor data were then used to recalculate D and K maps, which are denoted by D” and K” respectively; **(7)** use D”, K” and baseline image to calculate DW images with nonzero b-values (again, using *Eq*. (2)) which forms our noise-free DKI phantom; **(8)** add noise to the noise-free phantom, Rician noise can be added using *Eq*. (3):

$$I_{n}=\sqrt{{\left(I+n_{1,\sigma }\right)}^{2}+n_{2,\sigma }^{2}}$$ (3)

where *n*_*1*,*σ*_ and *n*_*2*,*σ*_ are both Gaussian distributed noise with standard deviation of *σ*. A fixed *σ* is used for all DW images when noises are added. A discussion on noise adding can be found in Section of Discussion and Conclusion.

### Non-Local Means (NLM) Filter Family

NLM [24] is a spatial domain filter that replaces each pixel *P(i)* in the image with a weighted average of every pixel *P(j)* in its “search region” *Ω*:

$$NLM(P(i))=Z_{0}\sum_{\forall j\in \Omega }\omega (i,j)P(j)$$ (4)

where *Z*_*0*_ is the normalization coefficient, defined as:

$$Z_{0}=1/\sum_{\forall j\in \Omega }\omega (i,j)$$ (5)

The weight *ω(i*,*j)* assigned to *P(j)* is based on the weighted Euclidean distance *d* between the neighborhoods of pixels *i* and *j*, named *R*_*f*_ *(i)* and *R*_*f*_ *(j)* respectively:

$$d(i,j)=G_{\rho }{\left\|R_{f}(i)-R_{f}(j)\right\|}^{2},(i\neq j)$$ (6)

$$\omega (i,j)=\exp(-d(i,j)/h^{2}),(i\neq j)$$ (7)

where *h* is a parameter that controls the degree of smoothing and is normally set proportionally to the standard deviation of noise. *G*_*ρ*_ is a Gaussian kernel of standard deviation *ρ*. In theory, the search region *Ω* in *Eq*. (5) can cover the whole image (thus non-local). However, a limited radius t is commonly adopted with regard to computational efficiency [25], [38]. When calculating the weight of the center pixel itself, the distance is simply set to the minimum distance found in the search region.

Similar to other weighting average filters, larger weights are assigned to pixels with higher similarity. NLM is unique in that it uses the distance between neighborhoods of pixels instead of the distance between pixels themselves. Thus, it can make use of redundant information in texture patterns in the image for robust denoising.

Manjόn and his colleagues proposed an unbiased non-local means (UNLM) filter [38] to correct the gray level bias introduced by Rician noise that is typical in MR images [15] [16], [17]. UNLM subtracts the bias from the NLM filtered image, which can be expressed as:

$$UNLM(P(i))=\sqrt{{\left(NLM(P(i))\right)}^{2}-2\sigma_{r}^{2}}$$ (8)

where *σ*_*r*_ denotes the standard deviation of Rician noise. In this work, all algorithms involved in the comparison use bias subtraction as in UNLM.

Wiest et al. proposed the VNLM filter [29] to denoise the DTI dataset. As previously mentioned, DTI acquires DW images using at least six different diffusion directions. VNLM groups these images into a vector image and denoises the vector image as a whole. Therefore, the distance in NLM is redefined as the distance *d*_*v*_ between neighborhoods of two vectors:

$$d_{\nu }(i,j)=G_{\rho }{\sum_{\nu =1}^{V}\left\|R_{f,\nu }(i)-R_{f,\nu }(j)\right\|}^{2}/V,(i\neq j)$$ (9)

where *v* and *V* denote the index and total number of DW images, respectively.

We realize that when VNLM is applied to DKI dataset, there are two different ways to combine DW images into vector images. One is to combine images of the same b-value but of different directions of diffusion gradient as a vector (VNLM-b), and the other is to combine images of the same direction of diffusion gradient, but of different b-values (VNLM-d).

When applying NLM to MRI, Coupe et al. extended it to 3D [25], in which both the neighborhood window and the search region become cubes centered at the pixel in concern. While this makes better use of the redundant structure information in the 3D MRI data, parameters of the 3D NLM filter should be carefully set for balancing the denoising effect and computational efficiency [39].

## Experiments and Results

We adopted 3D NLM-based filters in the evaluation. Regarding the parameter setting, previous work showed that no significant improvement can be achieved with a search region greater than 11 × 11 = 121 pixels for a 2D NLM filter [38], and using a larger search region will significantly increase computational time. Thus, we adopted a 5 × 5 × 5 search region (125 voxels in 3D instead of 121 in 2D case), which is the same parameter used in previous work [39]. In addition, a 3 × 3 × 3 neighborhood window was employed in our work. A neighborhood window of such a size is common in 3D NLM processing [25], [39], which is normally smaller than the search region. The parameter *ρ* was set to commonly used value 1.0 [38]. The value of the parameter h for NLM, VNLM-b, and VNLM-d was set to 1.0*σ*, 0.8*σ*, and 1.2*σ* respectively according to their optimal performance based on an exhaustive search. The search result of NLM agrees with those in previous reports [25] [39] [40]. The standard deviation of noise *σ* in real data was calculated from a background region of the image. Assuming that the signal in the background region consists of only Rician noise, *σ* can be estimated from:

$$\sigma \text{=}\sqrt{\mu /2}$$ (10)

where *μ* is the mean value of squared signal intensity in the background region. A discussion on noise estimation can be found in Section of Discussion and Conclusion.

We used Mean Square Error (MSE), Bias and standard deviation (Std) for quantitative comparison of the denoising methods, which reflect precision and accuracy of the denoising methods by their definitions:

$$MSE=\frac{1}{N}\sum_{i}^{N}{\left(I_{i}-Q_{i}\right)}^{2}$$ (11)

$$Bias=\frac{1}{N}{\left\|\sum_{i}^{N}I_{i}-Q_{i}\right\|}_{1}$$ (12)

$$\text{Std}=\sqrt{\frac{1}{N}\sum_{i}^{N}{\left(I_{i}-\frac{1}{M}\sum_{\text{j}}^{M}I_{j}\right)}^{2}}$$ (13)

where *I* and *Q* denote noise-free and denoised image respectively, and *N* is the number of voxels. The MSE, Bias and Std are calculated only in the brain region.

### DKI Phantom Construction

**Fig 2:**
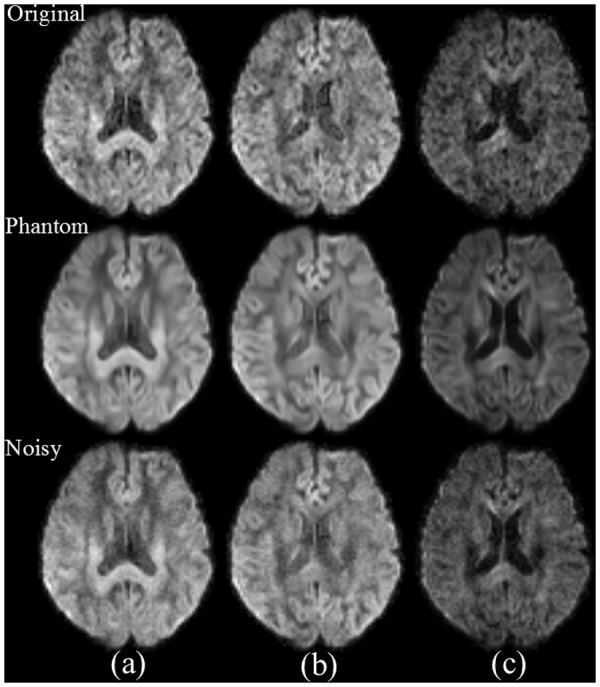
Results of DKI phantom construction. Top row: original noisy DW images; middle row: DW images of the constructed phantom; bottom row: DW images of the phantom, with synthetic Rician noise (σ = 10). The phantom consists of 151 volumes of DW images, including one baseline and 5 nonzero b-values (b = 500 ~ 2500 s/mm^2^) for each of 30 different diffusion gradient directions. Parameters of DW images are as follows: (a) b = 1000 s/mm^2^, at 16^th^ gradient direction, (b) b = 1000 s/mm^2^ at 1^st^ gradient direction and (c) b = 2000 s/mm^2^ at 1^st^ gradient direction.

We created a DKI phantom from Dataset 1 using the aforementioned process (Fig. 1). Compared with the original images, the constructed phantom is visually cleaner. In the phantom, noise is successfully suppressed, and anatomical structures are well preserved (Fig. 2).

### Filter Comparison with DKI Phantom

We compared the three NLM-based denoising algorithms mentioned earlier, NLM, VNLM-b and VNLM-d. First, five different levels of Rician noise (with standard deviations at 5, 10, 15, 20, 25) were added to DW images of the noise-free phantom. The resulting SNRs are different for DW images with distinct b-values, which are approximately 60, 30, 20, 15, 12 for baseline image (b = 0), or 12, 6, 4, 3, 2 for DW images with b = 2500 s/mm^2^. The noise-corrupted images were then denoised using the three filters, and results were evaluated both visually and quantitatively using the phantom and real dataset. Quantitative comparisons can be performed in multiple ways. For example, the denoised DW images can be compared with phantom DW images by calculating the MSE between them. Parameter maps calculated from denoised DW images can also be compared with those of the phantom. The latter approach allows a more comprehensive assessment of the filters with respect to parameter evaluation and should be favored in cases like DKI, in which producing a reliable parameter map is often the target of denoising. In this study, MD and MK maps, which were calculated by averaging D and K maps respectively along all diffusion gradient directions, are used to evaluate the denoising results. For robust statistical results, we repeated the above process that adds noise, denoises, and conducts performance evaluation for 500 times.

**Fig 3:**
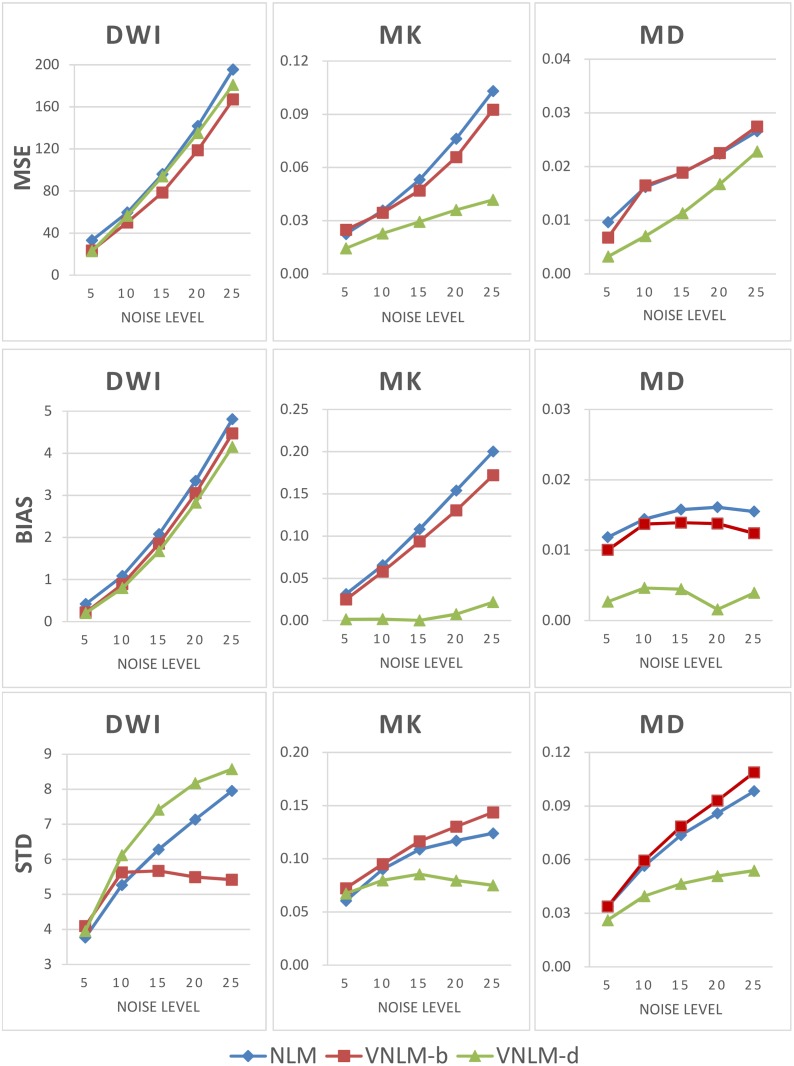
Quantitative comparison of NLM, VNLM-b and VNLM-d filters using the DKI phantom. Five levels of Rician noises with standard deviations of 5, 10, 15, 20, and 25 were added to the phantom DW images. The MSE, Bias and Std values of denoised DW images and MK and MD (DKI parameters) maps were calculated and compared. Top row: MSE value, middle row: Bias value, and bottom row: Std value.

The quantitative comparison of MD and MK maps shows that VNLM-d filter achieves lower MSE, Bias and Std than the original NLM and VNLM-b filters at almost all noise levels (Fig. 3). Moreover, the MSE, Bias and Std values of MK produced by the VNLM-d filter increase most slowly with the increase of noise level. When standard deviation of noise reaches 25, the MSE, Bias and Std values from the VNLM-d filter are only 40.6% (MSE), 10.9% (Bias) and 60.6% (Std) respectively of those from the NLM filter.

Meanwhile, it is interesting that comparing with the other two filters, the VNLM-d filter shows equivalent or even poorer performances concerning MSE and Std of the denoised DW images, which is contrary to its good performance for MD and MK maps as discussed above.

**Fig 4:**
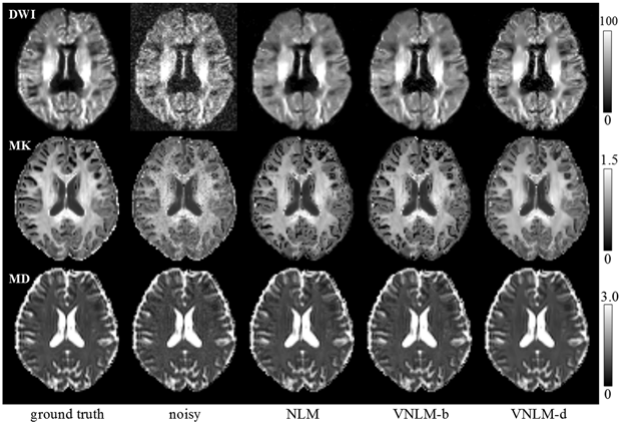
Visual comparison of denoising filters using the DKI phantom. Top row: DW images of b = 2500 s/mm^2^; middle row: MK maps; bottom row: MD maps. Images from left to right are ground truth, noisy image (with Rician noise of standard deviation = 10), and results from NLM, VNLM-b and VNLM-d filters.

A similar conclusion can be drawn from visual comparison. For MK maps, results from these three filters have different visual appearances. MK maps of NLM and VNLM-b filters contain black holes, which represent incorrectly estimated voxels [36], [41]. The MK map from VNLM-d filter is almost free of black holes and has a consistent visual appearance with the phantom MK map (see middle row of Fig. 4). Moreover, the DW image denoised by the VNLM-d filter show clearer those finer structural details than do those denoised by NLM and VNLM-b filters (see upper row of Fig. 4). The VNLM-d filter produces visually satisfying results even for low SNR DW images acquired with b = 2500 s/mm^2^.

### Denoising of Real dataset

To further validate the observation obtained from the phantom dataset, we also applied the NLM, VNLM-b and VNLM-d algorithms to Dataset 2 and evaluated the results for their performance. The parameters to these algorithms were set the same as described previously.

**Fig 5:**
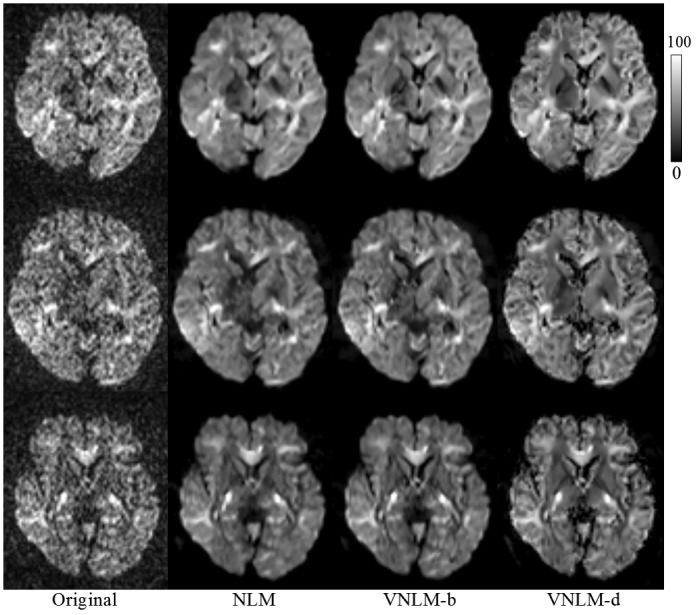
Comparison of denoising filters based on DW images (b = 2500 s/mm^2^) from real DKI dataset. Dataset of three volunteers are shown from top to bottom. Images from left to right are the original acquired images, and denoised results from NLM, VNLM-b, and VNLM-d filters.

**Fig 6:**
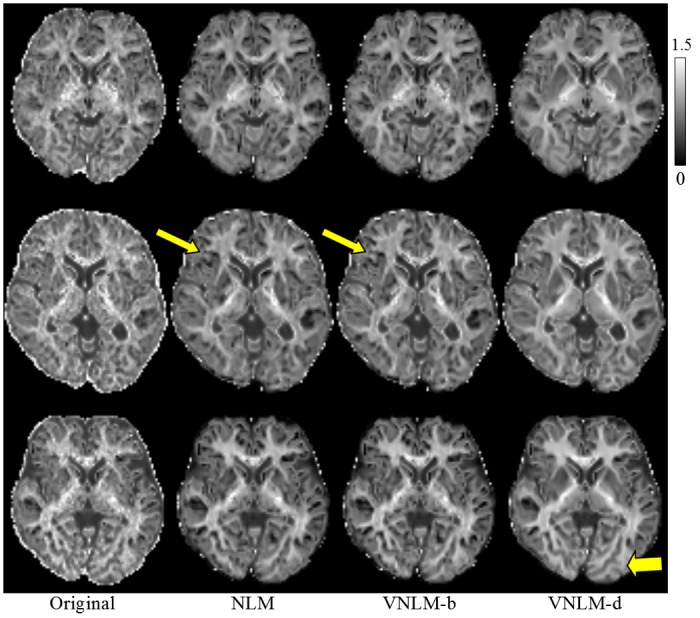
Comparison of denoising filters based on MK maps. From visual observation, The MK map from VNLM-d filter is almost free of black holes. The narrow arrows show the regions that more black holes are produced by NLM and VNLM-b filters. The black hole represents incorrectly estimated voxels. The bold arrow shows the more structural details preserved by VNLM-d filter.

**Fig 7:**
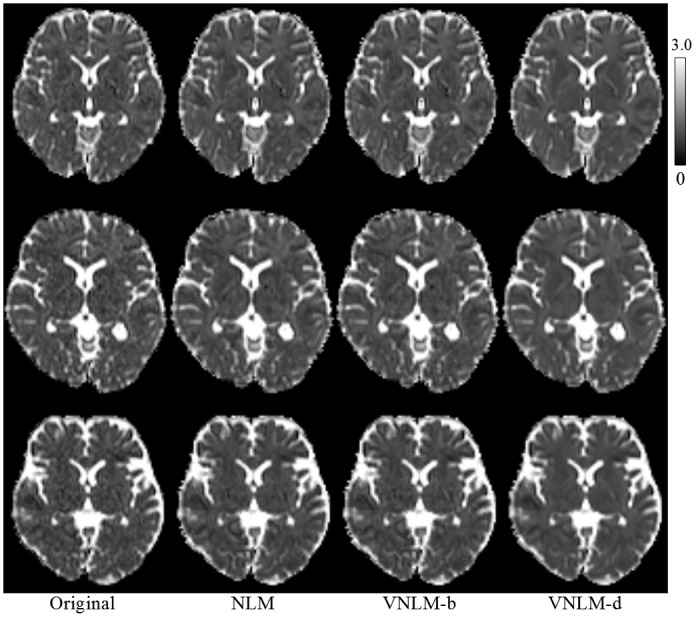
Comparison of denoising filters based on MD maps.

Visual comparison of the real dataset has revealed an impression similar to the information shown from the simulation data (Figs. 5–7). While all filters can significantly reduce noise in the DW images and parameter maps, VNLM-d produces DW images with clearer brain structure and MK map with fewer black holes for all three volunteers. In addition, we may see that the MK map provides microstructural information in gray matter (Fig. 6), which is not visible in MD (Fig. 7) or fractional anisotropy (FA) maps of diffusion tensor imaging model.

## Discussion and Conclusion

In this work, we propose a processing pipeline for constructing DKI phantoms using datasets of real human brains. The pipeline produces a high-SNR DKI phantom with clear image structure that can be used as ground truth for evaluation of DKI denoising methods. In addition, the proposed pipeline can also be used to generate a DKI dataset with a customized combination of b-values and diffusion gradient directions, different from those used in acquisition. This flexibility will be useful to evaluate the performance of various DKI acquisition schemes, such as 3-b-value fast scheme and also non-uniform schemes with different gradient directions at each b-value [42]. For these purposes, after the diffusion tensors and kurtosis tensors have been calculated in step (6), we can first apply a customized gradient table to recalculate D and K maps at the specified directions. DW images with desired b-values can then be calculated with D, K maps and baseline images using *Eq*. (2).

We should pointed out that Rician noise was used in step (8), because magnitude of MR signal is intrinsically corrupted by Rician noise, which is a model frequently adopted in MRI denoising and parameter estimation studies [15], [16], [17]. Nevertheless, the potential use of our DKI phantom is not limited to removal of Rician noise, as discussed above.

Furthermore, we quantitatively evaluated three NLM-based denoising algorithms using the constructed phantom. The simulation based on our phantom indicates that VNLM-d outperforms NLM, and the VNLM-b algorithms, generating more reliable MK and MD maps, with the lowest MSE, Bias and Std values for most of the noise levels. Visual comparison of these filters using a real dataset (Figs. 5–7) produces results consistent with this conclusion. While compared with the other methods, VNLM-d algorithm produces DW images with equal or higher MSE and Std (Fig. 3). This can be explained by the fact that VNLM-d tends to smooth more conservatively the structure in gray matter regions of the DW images. This lowers the level of image denoising but preserves more fine structures (Figs. 4, 5) which are helpful in the following parameter evaluation process. Thus, it yields less black hole effects and preserves structure better in MK maps (Fig. 6).

We think the good performance of VNLM-d filter can be attributed to the reason that the DKI parameter is calculated from diffusion decay curve, which means similar shape of the decay curves generate similar DKI parameters. VNLM-d filter treats DW images of different b-values at one direction as a unit in similarity calculation, thus voxels with similar decay curves, which means similar DKI parameters, tend to contribute more in the weighted average process. This efficiently exploits the similarity of DKI parameters.

**Fig 8:**
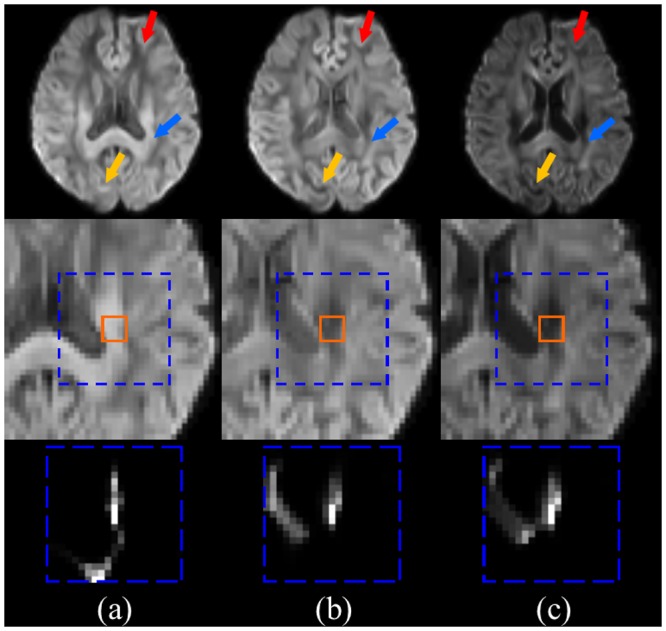
Comparison of structural similarities between DW images based on DKI phantom. From left to right, DW images acquired with (a) b = 1000 s/mm^2^, at 16^th^ gradient direction, (b) b = 1000 s/mm^2^ at 1^st^ gradient direction and (c) b = 2000 s/mm^2^ at 1^st^ gradient direction. The top and middle rows show the DW images and their corresponding magnified local regions, respectively; the bottom row shows NLM weight of a center voxel calculated in its surrounding search region. The solid line squares represent neighborhood windows of the center voxel, and the dashed line squares represent respective search regions. (b) and (c) are DW images acquired at the same gradient direction. Although their intensity and contrast is quite different, they have a greater structure similarity. The arrows indicate regions where (b) and (c) show similar structures, but (a) does not.

The structural preserving ability of VNLM-d filter may be attributed to the higher level of structural similarities between DW images, because VNLM is effective only when images with similar structures are grouped together, which have similar weights for averaging. We found DW images along the same direction but at different b-values (Fig. 8, column b and c) generally demonstrate higher similarities than do the DW images acquired at the same b-value but along different directions (Fig. 8, column a and b). Although the voxel intensities of DW images at the same b-values fall in a similar value range and are therefore more consistent with one another, it is the similarity between structures that leads to the superior performance of VNLM.

To further improve this work, several considerations can be taken into account. Firstly, DW images are often acquired with partial Fourier techniques, which can produce correlation between noises in neighboring pixels. This may present a new challenge to the denoising algorithms because many algorithms make an assumption of non-correlated noise. Despite of this, NLM filter has already been successfully applied to denoising DW images [17] [39]. This is understandable since when NLM calculates the weighted average to denoise a pixel, it considers the similarity between the neighborhood window of this pixel and those of the contributing pixels. The correlation between noises in neighboring pixels does not necessarily lead to similar neighborhood of these pixels, thus NLM filters are more robust to process correlated noise. Recently, NLM filter has also been improved to address correlated noise[43], and it is also suggested that denoising filter be used before partial Fourier reconstruction is carried out to avoid correlated noise [44]. Incorporating these results into our work may produce better results.

Secondly, in the procedures of adding and estimating noise, we assumed that the levels, spatial and statistical distributions of noise are all the same for all DW images at different b-values and along different diffusion gradient directions, which has been a commonly adopted hypothesis in previous studies [31] [34] [39]. However, this assumption may become invalid in certain cases. For example, the eddy current and off-resonance effects in a DWI sequence may potentially affect the noise, and these effects may substantially vary with b-value and diffusion gradient direction. In addition, the spatial and statistical distribution of noise can also be affected by the use of multi-element surface coils and parallel imaging [45]. For example, the noise distribution of parallel imaging is associated with a geometry factor (g-factor), which depends on coil geometry, phase-encoding direction and its acceleration factor [45] [46]. Thus, to more accurately evaluating the effects of simulating and estimating the noise in DW images, we must do a more careful simulation and inspect the influences that may be imposed by these factors. Limited by the length of this paper, and considering that this paper’s focus on reporting the general framework of providing a DKI phantom system, we decided not to pursue it in this study but to include it in our next step work.

Thirdly, motion artifacts, eddy current and geometrical distortion are major challenges in preprocessing of diffusion imaging data. Navigator based methods [47] [48] can be used to minimize motion artifacts prospectively. Multi-shot EPI [49] or fast spin echo sequences [50] can also be used to reduce geometry distortion due to susceptibility changes at tissue interfaces. Thus these methods should be considered in our further work to improve the quality of DKI estimation. In our constructed phantom data, signal dropout can be found in medial frontal and bilateral gray matter (Fig. 4). We think this can be attributed to individual variations that this particular individual happened to introduce some motion between the averages of data, and consequently motion artifacts. Retrospective Motion correction may not completely eliminated these artifacts.

## References

[1] Jensen JH, Helpern JA, Ramani A, Lu H, Kaczynski K (2005) Diffusional kurtosis imaging: the quantification of non-gaussian water diffusion by means of magnetic resonance imaging. Magn Reson Med 53: 1432–1440. 15906300 10.1002/mrm.20508

[2] Basser PJ, Mattiello J, LeBihan D (1994) MR diffusion tensor spectroscopy and imaging. Biophys J 66: 259–267. 8130344 10.1016/S0006-3495(94)80775-1 PMC1275686

[3] Cheung MM, Hui ES, Chan KC, Helpern JA, Qi LQ, et al (2009) Does diffusion kurtosis imaging lead to better neural tissue characterization? A rodent brain maturation study. NeuroImage 45: 386–392. 10.1016/j.neuroimage.2008.12.018 19150655

[4] Veraart J, Poot DHJ, Van Hecke W, Blockx I, Van der Linden A, et al (2011) More Accurate Estimation of Diffusion Tensor Parameters Using Diffusion Kurtosis Imaging. Magnetic Resonance in Medicine 65: 138–145. 10.1002/mrm.22603 20878760

[5] Lu H, Jensen JH, Ramani A, Helpern JA (2006) Three-dimensional characterization of non-gaussian water diffusion in humans using diffusion kurtosis imaging. NMR Biomed 19: 236–247. 16521095 10.1002/nbm.1020

[6] Wu EX, Cheung MM (2010) MR diffusion kurtosis imaging for neural tissue characterization. NMR Biomed 23: 836–848. 10.1002/nbm.1506 20623793

[7] Jensen CR (2011) White Matter Characterization with Diffusional Kurtosis Imaging. Neuroimage. 10.1016/j.neuroimage.2011.06.006 PMC3136876 21699989

[8] Wang JJ, Lin WY, Lu CS, Weng YH, Ng SH, et al (2011) Parkinson disease: diagnostic utility of diffusion kurtosis imaging. Radiology 261: 210–217. 10.1148/radiol.11102277 21771952

[9] Blockx I, De Groof G, Verhoye L, Van Audekerke J, Raber K, et al (2012) Microstructural changes observed with DKI in a transgenic Huntington rat model: Evidence for abnormal neurodevelopment. NeuroImage 59: 957–967. 10.1016/j.neuroimage.2011.08.062 21906685

[10] Yuzhen Zhang XY, Gao Y, Xu D, Wu J, Li Y (2013) A Preliminary Study of Epilepsy in Children Using Diffusional Kurtosis Imaging. Clinical Neuroradiology 23: 293–300. 10.1007/s00062-013-0212-3 23715877

[11] Falangola MF, Jensen JH, Babb JS, Hu C, Castellanos FX, et al (2008) Age-related non-Gaussian diffusion patterns in the prefrontal brain. J Magn Reson Imaging 28: 1345–1350. 10.1002/jmri.21604 19025941 PMC2669671

[12] Helpern JA, Adisetiyo V, Falangola MF, Hu C, Di Martino A, et al (2011) Preliminary evidence of altered gray and white matter microstructural development in the frontal lobe of adolescents with attention-deficit hyperactivity disorder: a diffusional kurtosis imaging study. J Magn Reson Imaging 33: 17–23. 10.1002/jmri.22397 21182116 PMC3492944

[13] Raab P, Hattingen E, Franz K, Zanella FE, Lanfermann H (2010) Cerebral gliomas: diffusional kurtosis imaging analysis of microstructural differences. Radiology 254: 876–881. 10.1148/radiol.09090819 20089718

[14] Yan X, Zhou M, Ying L, Liu W, Yang G, et al (2014) A fast schema for parameter estimation in diffusion kurtosis imaging. Comput Med Imaging Graph 38: 469–480. 10.1016/j.compmedimag.2014.06.010 25016957 PMC4150694

[15] Veraart J, Van Hecke W, Sijbers J (2011) Constrained maximum likelihood estimation of the diffusion kurtosis tensor using a Rician noise model. Magn Reson Med 66: 678–686. 10.1002/mrm.22835 21416503

[16] Sijbers J, den Dekker AJ (2004) Maximum likelihood estimation of signal amplitude and noise variance from MR data. Magn Reson Med 51: 586–594. 15004801 10.1002/mrm.10728

[17] Wiest-Daessle N, Prima S, Coupe P, Morrissey SP, Barillot C (2008) Rician noise removal by non-Local Means filtering for low signal-to-noise ratio MRI: applications to DT-MRI. Med Image Comput Comput Assist Interv 11: 171–179. 18982603 10.1007/978-3-540-85990-1_21 PMC2665702

[18] Brion V, Poupon C, Riff O, Aja-Fernandez S, Tristan-Vega A, et al (2011) Parallel MRI noise correction: an extension of the LMMSE to non central chi distributions. Med Image Comput Comput Assist Interv 14: 226–233. 21995033 10.1007/978-3-642-23629-7_28

[19] Parker GJ, Schnabel JA, Symms MR, Werring DJ, Barker GJ (2000) Nonlinear smoothing for reduction of systematic and random errors in diffusion tensor imaging. J Magn Reson Imaging 11: 702–710. 10862071 10.1002/1522-2586(200006)11:6<702::aid-jmri18>3.0.co;2-a

[20] Pennec X, Fillard P, Ayache N (2006) A Riemannian framework for tensor computing. International Journal of Computer Vision 66: 41–66.

[21] Tabelow K, Polzehl J, Spokoiny V, Voss HU (2008) Diffusion tensor imaging: structural adaptive smoothing. Neuroimage 39: 1763–1773. 18060811 10.1016/j.neuroimage.2007.10.024

[22] Ding Z, Gore JC, Anderson AW (2005) Reduction of noise in diffusion tensor images using anisotropic smoothing. Magn Reson Med 53: 485–490. 15678537 10.1002/mrm.20339

[23] Aja-Fernandez S, Niethammer M, Kubicki M, Shenton ME, Westin CF (2008) Restoration of DWI data using a Rician LMMSE estimator. IEEE Transactions on Medical Imaging 27: 1389–1403. 10.1109/TMI.2008.920609 18815091 PMC2756835

[24] Buades A, Coll B, Morel JM (2005) A Review of Image Denoising Algorithms, with a New One. Multiscale Model Simul 4: 490–530.

[25] Coupe P, Yger P, Barillot C (2006) Fast non local means denoising for 3D MR images. Med Image Comput Comput Assist Interv Int Conf Med Image Comput Comput Assist Interv 9: 33–40. 10.1007/11866763_5 17354753

[26] Naegel B, Cernicanu A, Hyacinthe JN, Tognolini M, Vallee JP (2009) SNR enhancement of highly-accelerated real-time cardiac MRI acquisitions based on non-local means algorithm. Med Image Anal 13: 598–608. 10.1016/j.media.2009.05.006 19541530

[27] Manjon JV, Coupe P, Marti-Bonmati L, Collins DL, Robles M (2010) Adaptive non-local means denoising of MR images with spatially varying noise levels. J Magn Reson Imaging 31: 192–203. 10.1002/jmri.22003 20027588

[28] Liu H, Yang C, Pan N, Song E, Green R (2010) Denoising 3D MR images by the enhanced non-local means filter for Rician noise. Magn Reson Imaging 28: 1485–1496. 10.1016/j.mri.2010.06.023 20850239

[29] Wiest-Daessle N, Prima S, Coupe P, Morrissey SP, Barillot C (2007) Non-local means variants for denoising of diffusion-weighted and diffusion tensor MRI. Med Image Comput Comput Assist Interv 10: 344–351. 18044587 10.1007/978-3-540-75759-7_42 PMC2129122

[30] Van Hecke W, Leemans A, De Backer S, Jeurissen B, Parizel PM, et al (2010) Comparing isotropic and anisotropic smoothing for voxel-based DTI analyses: A simulation study. Hum Brain Mapp 31: 98–114. 10.1002/hbm.20848 19593775 PMC6871062

[31] Basu S, Fletcher T, Whitaker R (2006) Rician noise removal in diffusion tensor MRI. Med Image Comput Comput Assist Interv Int Conf Med Image Comput Comput Assist Interv 9: 117–125. 10.1007/11866565_15 17354881

[32] Manjon JV, Thacker NA, Lull JJ, Garcia-Marti G, Marti-Bonmati L, et al (2009) Multicomponent MR Image Denoising. Int J Biomed Imaging 2009: 756897 10.1155/2009/756897 19888431 PMC2771160

[33] Tristan-Vega A, Aja-Fernandez S (2009) Design and construction of a realistic DWI phantom for filtering performance assessment. Med Image Comput Comput Assist Interv 12: 951–958. 20426080 10.1007/978-3-642-04268-3_117

[34] Van Hecke W, Sijbers J, De Backer S, Poot D, Parizel PM, et al (2009) On the construction of a ground truth framework for evaluating voxel-based diffusion tensor MRI analysis methods. Neuroimage 46: 692–707. 10.1016/j.neuroimage.2009.02.032 19268708

[35] Bansal R, Staib LH, Xu D, Laine AF, Liu J, et al (2009) Using Perturbation theory to reduce noise in diffusion tensor fields. Med Image Anal 13: 580–597. 10.1016/j.media.2009.05.001 19540791 PMC2782748

[36] Yan X, Zhou M, Ying L, Yin D, Fan M, et al (2013) Evaluation of optimized b-value sampling schemas for diffusion kurtosis imaging with an application to stroke patient data. Comput Med Imaging Graph 37: 272–280. 10.1016/j.compmedimag.2013.04.007 23735303 PMC3799948

[37] Reese TG, Heid O, Weisskoff RM, Wedeen VJ (2003) Reduction of eddy-current-induced distortion in diffusion MRI using a twice-refocused spin echo. Magn Reson Med 49: 177–182. 12509835 10.1002/mrm.10308

[38] Manjon JV, Carbonell-Caballero J, Lull JJ, Garcia-Marti G, Marti-Bonmati L, et al (2008) MRI denoising using non-local means. Med Image Anal 12: 514–523. 10.1016/j.media.2008.02.004 18381247

[39] Tristan-Vega A, Aja-Fernandez S (2010) DWI filtering using joint information for DTI and HARDI. Med Image Anal 14: 205–218. 10.1016/j.media.2009.11.001 20005152

[40] Coupe P, Yger P, Prima S, Hellier P, Kervrann C, et al (2008) An optimized blockwise nonlocal means denoising filter for 3-D magnetic resonance images. IEEE Trans Med Imaging 27: 425–441. 10.1109/TMI.2007.906087 18390341 PMC2881565

[41] Tabesh A, Jensen JH, Ardekani BA, Helpern JA (2011) Estimation of tensors and tensor-derived measures in diffusional kurtosis imaging. Magn Reson Med 65: 823–836. 10.1002/mrm.22655 21337412 PMC3042509

[42] Poot DHJ, den Dekker AJ, Verhoye M, Blockx I, Van Audekerke J, et al (2009) Optimizing the diffusion weighting gradients for diffusion-kurtosis imaging. Proc Intl Soc Mag Reson Med 17: 1394–1394.

[43] Aelterman J, Goossens B, Pizurica A, Philips W (2009) Suppression of Correlated Noise, Recent Advances in Signal Processing; Zaher AA, editor: InTech 10.14219/jada.archive.2009.0034

[44] Haldar JP, Wedeen VJ, Nezamzadeh M, Dai G, Weiner MW, et al (2013) Improved diffusion imaging through SNR-enhancing joint reconstruction. Magn Reson Med 69: 277–289. 10.1002/mrm.24229 22392528 PMC3407310

[45] Dietrich O, Raya JG, Reeder SB, Reiser MF, Schoenberg SO (2007) Measurement of signal-to-noise ratios in MR images: Influence of multichannel coils, parallel imaging, and reconstruction filters. Journal of Magnetic Resonance Imaging 26: 375–385. 17622966 10.1002/jmri.20969

[46] Goerner FL, Clarke GD (2011) Measuring signal-to-noise ratio in partially parallel imaging MRI. Medical Physics 38: 5049 10.1118/1.3618730 21978049 PMC3170395

[47] Alhamud A, Tisdall MD, Hess AT, Hasan KM, Meintjes EM, et al (2012) Volumetric navigators for real-time motion correction in diffusion tensor imaging. Magn Reson Med 68: 1097–1108. 10.1002/mrm.23314 22246720 PMC3330197

[48] Aksoy M, Forman C, Straka M, Skare S, Holdsworth S, et al (2011) Real-Time Optical Motion Correction for Diffusion Tensor Imaging. Magnetic Resonance in Medicine 66: 366–378. 10.1002/mrm.22787 21432898 PMC3139706

[49] Porter DA, Heidemann RM (2009) High resolution diffusion-weighted imaging using readout-segmented echo-planar imaging, parallel imaging and a two-dimensional navigator-based reacquisition. Magn Reson Med 62: 468–475. 10.1002/mrm.22024 19449372

[50] Xu D, Henry RG, Mukherjee P, Carvajal L, Miller SP, et al (2004) Single-shot fast spin-echo diffusion tensor imaging of the brain and spine with head and phased array coils at 1.5 T and 3.0 T. Magn Reson Imaging 22: 751–759. 15234443 10.1016/j.mri.2004.01.075

